# The Difference in Cervical Vertebral Skeletal Maturation between Cleft Lip/Palate and Non-Cleft Lip/Palate Orthodontic Patients

**DOI:** 10.1155/2018/5405376

**Published:** 2018-01-28

**Authors:** Waeil Batwa, Khalid Almoammar, Aziza Aljohar, Abdullah Alhussein, Saad Almujel, Khalid H. Zawawi

**Affiliations:** ^1^Department of Orthodontics, Faculty of Dentistry, King Abdulaziz University, Jeddah, Saudi Arabia; ^2^Division of Orthodontics, Department of Pediatric Dentistry and Orthodontics, College of Dentistry, King Saud University, Riyadh, Saudi Arabia; ^3^Division of Orthodontics, Department of Dentistry, King Faisal Specialist Hospital & Research Center, Riyadh, Saudi Arabia; ^4^College of Dentistry, King Saud University, Riyadh, Saudi Arabia

## Abstract

**Objective:**

The aim was to evaluate differences in the cervical vertebral skeletal maturity of unilateral cleft lip and palate (UCLP) and non-cleft lip/palate (non-CLP) Saudi male orthodontic patients.

**Method:**

This cross-sectional multicenter study took place at the dental school, King Saud University and King Faisal Specialist Hospital and Research Center, Riyadh, Saudi Arabia, between October 2014 and September 2015. The records of Saudi male orthodontic patients with UCLP (*n* = 69) were collected. Cervical vertebral maturation was assessed using their cephalometric radiographs. The records of 138 age-matched non-CLP Saudi male orthodontic patients served as controls.

**Results:**

There was a significant difference in skeletal maturity between the UCLP and non-CLP groups, as evident in the delayed skeletal development among the UCLP participants. Moreover, pubertal growth spurt onset was significantly earlier in the non-cleft participants in comparison with the UCLP participants (*p* = 0.009).

**Conclusions:**

There is delayed skeletal maturity among the UCLP Saudi male population in comparison with their non-CLP age-matched peers.

## 1. Introduction

Cleft lip or palate (or both) is considered a common congenital facial malformation, and its prevalence in Saudi Arabia ranges from 0.3 to 2.19 per 1000 live births [1, 2]. In addition to their social difficulties [3], children with a cleft lip and/or palate (CLP) inherit multiple complications related to inadequate nutrition, feeding problems [4], and speech impairment [5]. The literature has shown that growth in general [4, 6] and craniofacial complex growth in particular could be affected in children with CLP, leading to marked skeletal discrepancies in all three planes of space [7]. Other complications include several occlusal and dental discrepancies [7]. Understanding craniofacial growth and development is essential for the comprehensive and successful management of these orthodontic patients. Such knowledge plays a crucial role in the diagnosis, treatment planning, outcomes, and overall stability of patient's orthodontic treatment [8]. Cleft patient treatment aims to address skeletal and dental disharmony through multidisciplinary care, where skeletal discrepancies in children with CLP may require orthopedic and/or surgical correction [9].

Generally, orthodontic treatment and intervention are timed to take place before or during the peak growth velocity or pubertal growth spurt to achieve favorable effects in correcting sagittal, transverse, and vertical plane disharmonies [10, 11]. Skeletal maturity and growth spurts have been assessed by several methods in the literature, including chronological age, dental development, and sexual maturation characteristics [12]. All of these methods have limitations, such as poor correlation with growth spurt.

**Figure 1:**
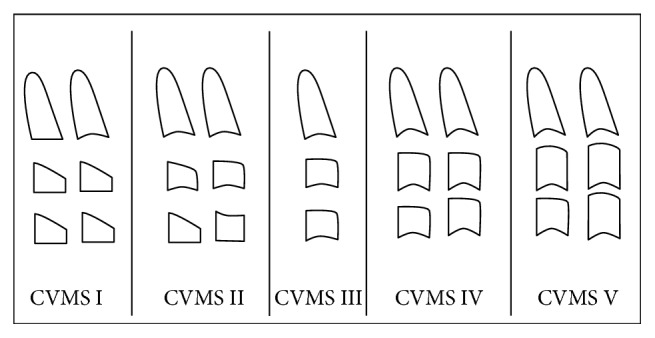
The modified five stages of cervical vertebral maturation [10].

Other more accurate measures include growth charts and skeletal age [12, 13]. Hand-wrist radiographs for assessing skeletal maturity show good correlation to the growth velocity of the face [14]. An alternative process, referred to as the cervical vertebral maturation (CVM) method, uses lateral cephalometric radiography to evaluate skeletal maturity [10]. Due to its advantage of avoiding unnecessary extra radiation, the CVM method is currently considered preferable. Baccetti et al. modified the CVM method to include five maturation stages by evaluating vertebrae C2 to C4 [10] (Figure 1).

Moreover, ethnicity could influence the timing of skeletal maturation [12]. Since there is limited information about the skeletal development of many ethnic groups and populations including Saudis, this study aimed to evaluate the skeletal maturity of UCLP and non-CLP Saudi orthodontic male patients by judging the difference in CVM stages.

## 2. Materials and Methods

This cross-sectional multicenter study was carried out at the dental school, King Saud University (KSU) and King Faisal Specialist Hospital and Research Center (KFSH&RC), Riyadh, Saudi Arabia, between October 2014 and September 2015. The study was reviewed and approved by the Research Center of the College of Dentistry at KSU (number 3536024, IR0107).

The CLP databases at the College of Dentistry in KSU and KFSH&RC included a total of 469 CLP patients. Lateral cephalometric radiographs of UCLP and non-CLP (control group) male subjects were recruited, and the following criteria were applied: (1) Saudi patients with nonsyndromic UCLP, aged 10–16 years; (2) Saudi patients who are nonsyndromic, non-CLP, aged 10–16 years; (3) absence of any birth defects or any other anomalies that could alter skeletal growth; and (4) all included patients having complete records comprising dental/medical files and a lateral cephalometric radiograph.

The UCLP and the non-CLP groups were subdivided based on chronological age:

  Group 1C: UCLP participants from age 10 to 13.
  Group 1N: non-CLP participants from age 10 to 13.
  Group 2C: UCLP participants from age >13 to 16.
  Group 2N: non-CLP participants from age >13 to 16.

An experienced examiner performed the analysis of the lateral cephalometric radiographs manually using a viewing box with standardized settings in a darkroom. The evaluations of CVM were undertaken according to Baccetti et al.'s [10] method. Cervical vertebral maturation stage (CVMS) is identified by examining the C2, C3, and C4 vertebrae. When the lower borders of C2, C3, and C4 are flat—with a possible concavity at the lower border of C2—while the bodies of both C3 and C4 are trapezoidal in shape, CVMS I is indicated. When a concavity appears at the lower borders of C2 and C3 and the bodies of both C3 and C4 are either trapezoidal or rectangular horizontal in shape, CVMS II is indicated. In CVMS III, a concavity forms at the lower borders of C2, C3, and C4, while the bodies of C3 and C4 are horizontally rectangular. When a concavity is present at the lower borders of C2, C3, and C4 and the bodies of C3 and C4 become square, this indicates CVMS IV. The transition to CVMS V is identified when a concavity appears at C2, C3, and C4, where the bodies of C3 and/or C4 are vertically rectangular.

The shapes of the cervical vertebrae were visually analyzed according to Baccetti et al.'s definitions [10], as follows:

*Trapezoid* is identified when the upper border is pointed from the posterior to the anterior, while* rectangular horizontal* is when the posterior and anterior border heights are equal but shorter than the inferior and superior ones. Conversely,* rectangular vertical* is when the superior and inferior borders are shorter than the heights of the posterior and anterior borders. Finally,* squared* is when the all four borders are equal (Figure 1).

Intraexaminer reliability was tested by randomly selecting 27 lateral cephalometric radiographs and analyzing them for skeletal maturation on two separate occasions with a two-week interval. The kappa value was 0.921, which is considered excellent agreement.

### 2.1. Statistical Analysis

Categorical outcome and variables were explained using descriptive statistics (frequencies and percentages), and Student's *t*-test was used to compare the mean age between the UCLP and non-CLP groups. A *p* value of <0.05 was used to report the statistical significance of the results. All statistics were carried out with Statistical Package for Social Science Software (IBM SPSS Statistics for Windows, Version 21.0, Armonk, NY: IBM Corp, USA).

## 3. Results

In total, 69 UCLP male participants and 138 non-CLP male participants were included. The UCLP group consisted of 41 (59.4%) left-sided UCLP participants and 28 (40.6%) right-sided UCLP participants. Equal numbers of participants were recruited in each age range for the non-CLP group. However, for the UCLP group, the number varies between age ranges, with fewer patients aged 12-13 and 13-14 (Table 1).

Table 2 shows the distribution of patients among the four groups. In total, 36 and 33 participants were recruited to groups 1C and 2C, respectively, while 69 were recruited to each of groups 1N and 2N.

Most of the group 1C participants were at CVMS I (75%), but this was not the case for group 1N, where the participants were almost equally distributed between CVMS I and CVMS II (43.5% and 34.8%, respectively). For group 1C, 27 patients were at CVMS I while 6 patients were at CVMS II and 3 patients were at CVMS III; no patients had reached CVMS IV and CVMS V. Further, 30 patients of group 1N were at CVMS I, 24 were at CVMS II, 14 were at CVMS III, and 1 was at CVMS IV, while no patients were at CVMS V.

On the other hand, the majority of group 2C and 2N participants were at CVMS III and CVMS IV. In group 2C, 6 patients were at CVMS I, 4 were at CVMS II, 12 were at CVMS III, 7 were at CVMS IV, and 4 were at CVMS V. In group 2N, 1 patient was at CVMS I, 7 were at CVMS II, 33 were at CVMS III, 19 were at CVMS IV, and 9 were at CVMS V.

The counts of participants in groups 1C and 2C were combined for CVMS III only, to compare UCLP and non-CLP patients. The same was done for non-CLP patients. Stage CVMS III appeared at a significantly earlier age in the non-CLP group (mean = 13.86 ± 1.3 years) when compared to the UCLP group (mean = 12.95 ± 1.08 years), with a mean difference of 0.9 years (10.8 months). This difference was highly significant (*p* = 0.009) (Table 3), reflecting a delayed onset of the pubertal growth spurt among the UCLP participants in comparison with the non-CLP participants.

## 4. Discussion

There are substantial growth impairments and skeletal disproportions associated with the CLP anomaly [4, 6, 7]. Maxillary retrusion, class III skeletal disproportion, and crossbites (anterior and/or posterior) are common findings in CLP patients [7]. These malformations are linked to scars, numerous surgical procedures early in life, and/or a growth pattern that is usually altered in cleft participants because of structural malformation in the area of the oral cleft. One would expect to see anterior crossbite in patients with complete clefts who had been operated on in their childhood and in those with complete bilateral cleft lip and palate [15]. Individuals with only cleft lip and individuals with only cleft palate do not present anteroposterior maxillary growth deficiencies after cleft surgeries [16]. However, absence of midpalatal suture in patients with CLP can lead to transverse maxillary deficiencies [17] and posterior crossbite, and early palatoplasty magnifies this effect [18], causing further transverse maxillary deficiency; hence, these patients would benefit from expansion treatment of the permanent dentition [19]. In order to correct the posterior dental and skeletal crossbite (transverse problems), expansion via palatal expanders must be carried out before cessation of growth and preferably during the growth spurt peak. Therefore, it is of prime importance to study the skeletal maturity of CLP patients to predict their growth potential. Only a few studies have addressed skeletal maturation in CLP participants in comparison with a control population and their findings are limited to the gender and ethnicity studied [12, 20, 21].

The present cross-sectional retrospective study is the first to identify differences in skeletal maturation between nonsyndromic UCLP Saudi subjects and non-CLP subjects. Skeletal maturation, in this study, was assessed using the CVM method, as described by Baccetti et al. [10]. The CVM method is a valid, reliable, and reproducible method for assessing skeletal maturation and identifying the pubertal growth spurt [8, 10]. Using the current version of Baccetti et al. [10] made it easier for us to compare our findings with similar studies. Although digital radiograph tracing is easier and faster, manual assessment was found to be just as good with regard to its clinical value and acceptability [22, 23]. The identification of the pubertal growth spurt is deemed necessary when planning growth modification strategies to address skeletal discrepancies in growing participants. It is recommended that growth modification treatment coincides with CVMS III in male patients to make the best of the growth spurt [10, 24]. For these reasons, the participants in CVMS III in the CLP and non-CLP groups were compared. Ideally, this study should compare all CLP and non-CLP patients of both genders at all CVM stages, but the limited number of recruited participants, especially in the cleft group, forced us to focus only on the male patients and the most relevant stage for orthodontic treatment (CVMS III).

In the current study, the collected sample consisted of age-matched male UCLP and non-CLP participants ranging from 10 to 16 years. This ensured equal distribution agewise, which helped accurately identify the pubertal growth spurt in both groups. The wide age range was used deliberately to avoid overlooking any participants at CVMS III. Furthermore, dividing UCLP and non-CLP patients into four groups provided a better overview of the participant distribution among the CVM stages. The result showed that 75% of the UCLP participants in group C1 were at CVMS I, compared to only 43% in group N1. Certainly this is an indication that UCLP patients in group 1C have delayed skeletal maturity; however, it can be argued that this is due to the presence of more participants at age 10 than at age 13. Interestingly, a similar finding was observed for individuals in groups 2C and 2N, where some UCLP participants were at CVMS I and CVMS II (18.1% and 12.1%, respectively), with fewer participants in the non-CLP group at these stages (1.4% and 10.1%, respectively). This supports the suggestion that CLP patients experience more delayed skeletal development than non-CLP patients. The results revealed an overall delayed skeletal development among the UCLP participants in comparison with the non-CLP controls, which is consistent with the findings of Sun and Li male study [21]. On a different study, Sun and Li looked at Chinese female skeletal maturation and noticed a delay in skeletal maturity in cleft patients [20]; unfortunately this could not be compared to our sample due to gender influence on skeletal maturity, and future studies are recommended to study Saudi female skeletal development in cleft and non-cleft patients and compare it to male candidates and other ethnicities. Table 3 shows the CVMS for different ethnicity of matched ages. Moreover, it shows that skeletal maturity of Saudi and Indonesian patients is ahead of white people, with more patients recorded at CVMS I and CVMS II (Table 4).

Ideally, a sample size calculation should be carried out. However, due to the nature of the studied sample (UCLP) and the extreme difficulty of allocating a good number of patients with good records, even when we included two of the biggest cleft centers within the kingdom, we recruited a similar sample to Sun and Li, including all suitable allocated records [21]. Despite the smaller sample size of UCLP participants in the present study, the pattern of skeletal maturity and, in particular, the delayed skeletal development because of the UCLP anomaly were anticipated. When comparing our data or sample distribution at different CVM stages to the Chinese sample, the Chinese population was different, reaching its peak growth spurt before our group [12].

Our study included 10- to 16-year-old participants with an outcome that indicated a highly statistically significant difference between the UCLP and non-CLP patients of all ages at CVMS III (*p* = 0.009), confirming that participants with the UCLP anomaly are more likely to have a decelerated pubertal spurt and a delayed pubertal peak by almost 10 months. This proved the implication of UCLP anomalies in growth patterns, which has been proposed by many investigators [4, 6].

Even though this was a multicenter study, there are some limitations. The current study is cross-sectional; hence, clear conclusions can be drawn. Another limitation is that only male patients were selected. Future longitudinal multicenter studies with larger sample sizes including males and females are needed to confirm the current findings and establish the skeletal maturation of patients suffering from cleft lip/palate.

## 5. Conclusion

The skeletal maturity of Saudi male UCLP patients is significantly different compared to non-CLP age-matched peers. The non-CLP groups reached skeletal maturity before the UCLP groups. In addition, the onset of the pubertal growth spurt was significantly earlier in the non-CLP participants in comparison with the UCLP-affected participants. A larger sample size is required to draw a robust conclusion.

## Figures and Tables

**Table 1:** Distribution of patients by age range in the UCLP and non-CLP groups of Saudi male patients.

Patient age	UCLP	Non-CLP
10-11	14	23
11-12	15	23
12-13	7	23
13-14	5	23
14-15	12	23
15-16	16	23

Total	**69**	**138**

**Table 2:** The distribution of patients according to CVM stages (number and percentage) in Saudi male patients.

Ages	Group (*n*)	CVMS I	CVMS II	CVMS III	CVMS IV	CVMS V
10–13	UCLP (36)	27 (75%)	6 (16.6%)	3 (8.4%)	0 (0%)	0 (0%)
	Non-CLP (69)	30 (43.5%)	24 (34.8%)	14 (20.3%)	1 (1.4%)	0 (0%)
>13–16	UCLP (33)	6 (18.1%)	4 (12.1%)	12 (36.3%)	7 (21.2%)	4 (12.1%)
	Non-CLP (69)	1 (1.4%)	7 (10.1%)	33 (47.8%)	19 (27.5%)	9 (13.04%)

**Table 3:** Comparison of mean age of subjects at CVS 3 stage. Student's *t*-test was used to compare the mean age between the UCLP and non-CLP groups. A *p* value of <0.05 was used to report the statistical significance of the results.

Type of patients	Mean age	Mean difference	Standard deviation	Std error of mean	*t* value	*p* value
UCLP	13.86	0.9	1.30	0.33	2.695	0.009
Non-CLP	12.95		1.08	0.15		

**Table 4:** The distribution of patients of different ethnicity according to CVM stages (number and percentage) [12].

Ages	Group (*n*)	CVMS I	CVMS II	CVMS III	CVMS IV	CVMS V
10–16	Indonesian (1422)	522 (36.7%)	212 (14.9%)	385 (27%)	277 (19.5%)	26 (1.9%)
	White (745)	116 (15.6%)	85 (11.4%)	239 (32%)	253 (34%)	52 (7%)
	Saudi (138)	31 (22.5%)	31 (22.5%)	47 (34%)	20 (14.5%)	9 (6.5%)
